# Immersive Surgical Anatomy of the Pterional Approach

**DOI:** 10.7759/cureus.5216

**Published:** 2019-07-23

**Authors:** Roberto Rodriguez Rubio, Ricky Chae, Vera Vigo, Adib A Abla, Michael McDermott

**Affiliations:** 1 Neurological Surgery, University of California, San Francisco, USA

**Keywords:** surgical techniques, volumetric models, immersive, surgical neuroanatomy, pterional approach, minipterional, two-part pterional

## Abstract

The pterional approach (PA) is a versatile anterolateral neurosurgical technique that enables access to reach different structures contained in the cranial fossae. It is essential for neurosurgical practice to dominate and be familiarized with its multilayer anatomy. Recent advances in three-dimensional (3D) technology can be combined with dissections to better understand the spatial relationships between anatomical landmarks and neurovascular structures that are encountered during the surgical procedure. The present study aims to create a stereoscopic collection of volumetric models (VM) obtained from cadaveric dissections that depict the relevant anatomy and surgical techniques of the PA. Five embalmed heads and two dry skulls were used to record and simulate the PA. Relevant steps and anatomy of the PA were recorded using 3D scanning technology (e.g. photogrammetry, structured light scanner) to construct high-resolution VM. Stereoscopic images, videos, and VM were generated to demonstrate major anatomical landmarks for PA. Modifications of the standard PA, including the mini-pterional and two-part pterional approaches, were also described. The PA was divided into seven major steps: positioning, incision of the skin, dissection of skin flap, dissection of temporal fascia, craniotomy, drilling of basal structures, and dural opening. Emphasis was placed on preserving the temporal branches of the facial nerve and carefully dissecting the temporalis muscle. The interactive models presented in this article allow for clear visualization of the surgical anatomy and windows in 360-degrees and VR. This new modality of recording neuroanatomical dissections renders a closer look at every nuance of the topography experienced by our team in the laboratory. By accurately depicting essential landmarks, stereoscopy and VM can be valuable resources for anatomical education and surgical planning.

## Introduction

The pterional or frontotemporalsphenoidal craniotomy is an extensively used neurosurgical technique to expose the Sylvian fissure (SF). The pterional approach (PA) is performed around the pterion, which represents the intersection of the frontal, temporal, parietal, and sphenoid bones. Historically, the term is derived from the Greek root pteron and refers to the wings that were attached to the head of Hermes, the messenger of the Greek gods [1]. This approach aims to minimize brain retraction and increase the exposure of the neurovascular structures within the anterior basal cistern by carefully dissecting the temporalis muscle and drilling the sphenoid wing [2]. However, the surgeon must be cognizant of different landmarks that are encountered during dissections from the skin to the bone. Our collection relies on volumetric models (VMs) and stereoscopic media to effectively probe and understand the complex spatial relationships of neuroanatomy. By allowing users to actively interact with the structures, VMs represent a useful tool for anatomical education and surgical planning. The use of virtual reality (VR) and augmented reality (AR)-ready systems (e.g., computers and smartphones) are strongly recommended to have a fully immersive experience of the models included in this article.

## Technical report

**Video 1:** Volumetric model of the skull, with annotations of major ectocranial landmarks The following instructions can be used to manipulate all models: to move, left click and drag; to zoom in and out, use the mouse scroll. For smartphones and virtual reality (VR)-ready computers, click “view in VR” (glasses icon); to view annotations, click on the numbers, to move around the object tap or press trigger on the floor using the blinking yellow circle as a pointer. For mobile augmented reality (AR), click on the AR icon (cube) in the top right corner and aim at a horizontal flat surface; once the surface is detected tap on it to place the model.

Materials and Methods

Five embalmed and latex-injected cadaveric heads were prepared for surgical simulation along with two dry-skulls which were used to identify and documented the relevant osseous structures of the PA. Dissections were performed under a surgical simulation setting using a surgical microscope (OPMI® Pentero® 900, Zeiss, Germany) and stop-motion frames were recorded with a high-definition stereoscopic video device (Trenion™, Zeiss, Germany). Stereoscopic (side-by-side) pictures were taken using a professional camera (D810, Nikon, Japan) and selected specimens were prepared for two 3D scanning techniques (e.g., photogrammetry and structured light scanning). No IRB/ethics committee approval was required for this study.

Photogrammetry

Photos were taken of each cadaveric specimen at four angles in 360° with a professional camera (D810, Nikon, Japan) on a tripod (190x, Manfrotto, Italy) and an automatized turntable (Foldio360, Orangemonkie, USA). Batch image processing was performed using photography software (DxO Optics Pro 11, DxO, France) to adjusts exposure and enhance regions of interest. The images were then uploaded and rendered into a VM model using a photogrammetry software (Reality Capture BETA 1.0, Capturing Reality, Slovakia). This software superimposes the images and measures the intersecting tie points to triangulate the location of points in three-dimensional space and create a volumetric object. Visual improvements, namely decimation, smoothing, and texture adjustments, were made using a 3D computer graphics toolset (Blender 2.79, Netherlands).

Structured Light Scanning

Scans from 4 different positions (e.g., prone, supine, right lateral, and left lateral) were taken of a dry human skull specimen using a high-resolution 3D scanner based on blue light technology (Artec Space Spider, Artec 3D, Luxembourg). Frames of the scans were aligned, and a mesh was obtained to be subsequently texturized using the in-house software (Artec Studio 12, Artec, Luxembourg). Post-processing of the model (e.g., decimation, smoothing, texture adjustment) was performed using the toolkit included in the software of the 3D scanner.

Virtual Platform

VMs were uploaded to a web-based 3D model viewer app (Sketchfab, USA). Once the VMs were uploaded, the virtual scene was prepared for its real-time rendering. Position, lighting, materials, and filters were set to highlight regions of anatomical interest. Strategic points were labeled and annotated for an interactive experience. Views of the models were set for both 2D and 3D experiences.

Indications

**Figure 1:**
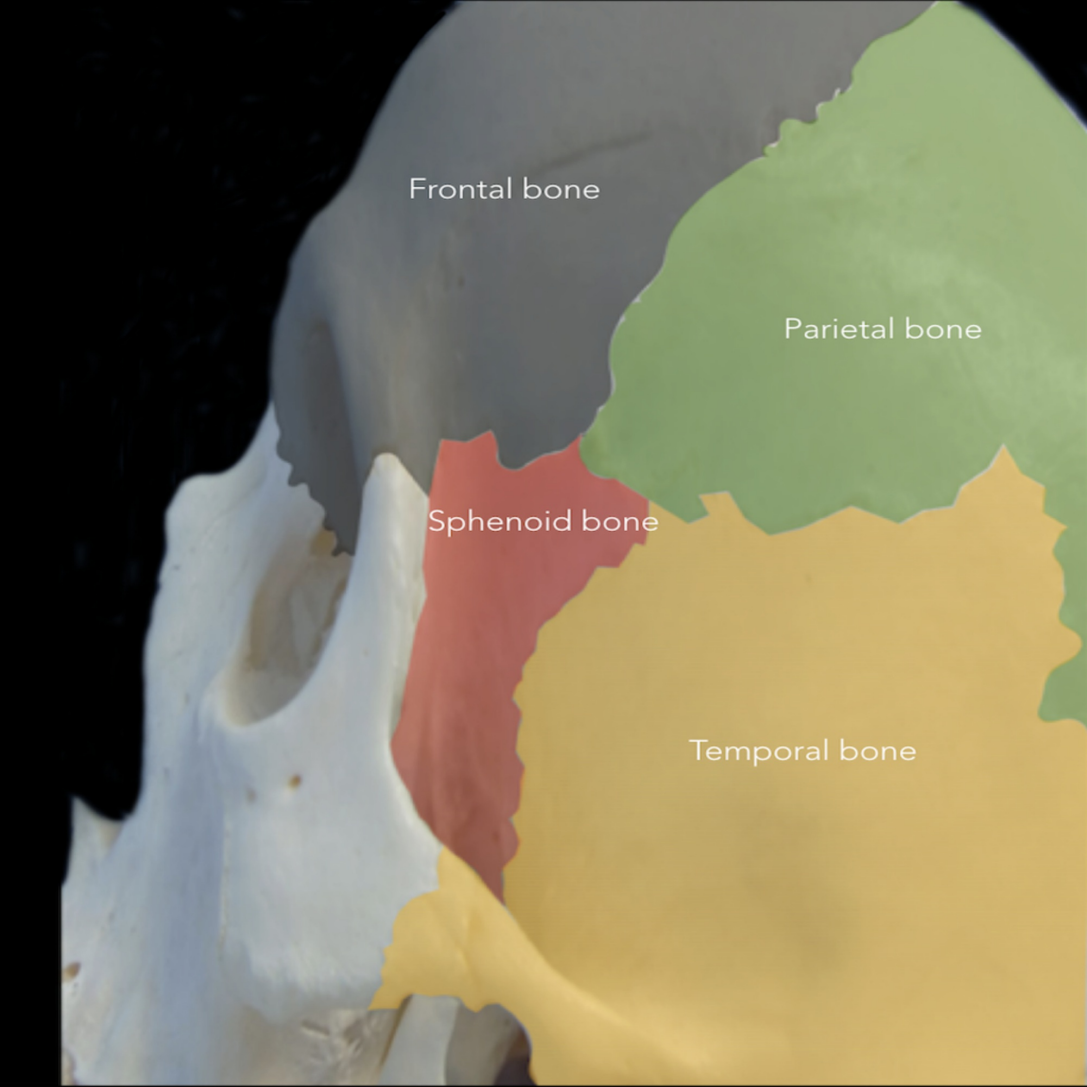
Cranial bones involved in the pterional approach The pterional approach (PA) is performed around the pterion, which represents the intersection of the frontal (grey), temporal (yellow), parietal (green), and sphenoid (red) bones.

**Figure 2:**
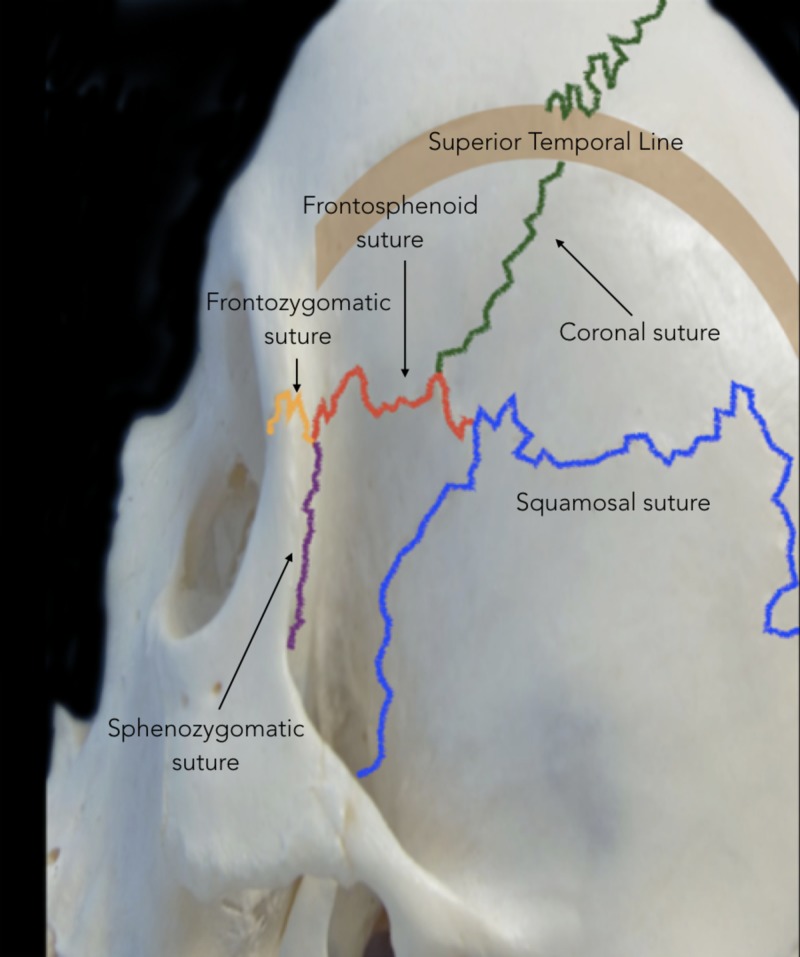
Cranial sutures involved in the pterional approach Lateral view showing cranial sutures relevant to the pterion: coronal suture (green), sphenofrontal suture (red), squamosal suture (blue), frontozygomatic suture (yellow), and sphenozygomatic (purple). The thick brown line represents the superior and inferior temporal lines.

The bony anatomy, including cranial sutures, should first be examined to supplement an understanding of the indications for PA (Figures 1, 2).

Major ectocranial landmarks can be visualized in VR for an immersive experience (Video 1; Interactive Models 1, 2).

**Video 1:** Virtual reality (360-degree stereoscopic) video showing a lateral-to-medial view of the cranial fossae The pterional craniotomy can be performed using various craniometric points, including the pterion (the intersection of the frontal, temporal, parietal, and sphenoid bones) and squamous suture. To view the video in virtual reality mode, Google Cardboard and YouTube mobile app are necessary. First, open the video on the YouTube mobile app and tap the Cardboard icon. Next, place the mobile device inside the Google Cardboard. Finally, look around to view the video in 360-degrees.

**Video 2:** Volumetric model of the skull base, with labels and annotations Various landmarks are shown for the anterior, middle, and posterior fossae.

Intracranially, the PA exposes the opercula and SF, which allows the surgeon to access the basal cistern, insula, basal ganglia, sellar and parasellar areas, hypothalamic area, third ventricle, anterior cranial fossa, anterior portion of the middle cranial fossa, and anterior and superior portions of the posterior cranial fossa (Interactive Model 3).

**Video 3:** Volumetric model of the pterional window, with labels and annotations The pterional approach provides access to structures of the anterior cranial fossa, anterior portion of the middle cranial fossa, and anterior and superior portions of the posterior cranial fossa.

Therefore, the PA can be used to reach aneurysms and other vascular lesions of the anterior circulation (internal carotid artery, anterior and middle cerebral arteries, posterior communicating artery); distal portion of the posterior circulation (basilar tip, first segment of posterior cerebral artery, superior cerebellar artery); tumors of the frontal, temporal, parietal, and insular lobes; and tumors of the orbit and anterior and middle skull base [1, 3, 4]. In selected cases, the surgical corridor of the PA can be extended contralaterally via arachnoidal dissection to reduce surgical time and morbidity [5]. Furthermore, this approach can be used to reach medial structures, such as the mesial temporal lobe, hippocampus, and upper brain stem [6].

Surgical Technique

Positioning

The patient should be positioned supine in a neutral position with a pad under the ipsilateral shoulder. The head should be lifted above the level of the heart to promote venous return and secured using a three-pin skull fixation device (Video 2).

**Video 2:** 3D (stereoscopic) video of head positioning for a pterional approach The patient’s head is elevated above the level of the heart, rotated 15 degrees away from the side of the aneurysm, and secured using a three-pin skull fixation device. The head is also extended 20 degrees to make the malar eminence the highest point in the surgical field.

The two contralateral pins should be positioned above the superior temporal line (STL) and the ipsilateral pin behind the incision line above the STL. The ipsilateral pin can be placed in the mastoid tip to reduce visual obstructions for the surgeon. Alternatively, two pins can be placed on the same side, posteriorly of the ear, and the contralateral pin on the frontal bone just above the hairline. The head should be rotated about 15-20 degrees away from the side of the lesion, which can be adjusted depending on the target. Finally, the head is extended approximately 20 degrees to make the malar eminence the highest point in the surgical field. These steps allow a natural separation of the frontal lobe and reduce the use of retractors.

Incision of skin

A curvilinear skin incision is made from the superior rim of the zygomatic arch, 1 cm anterior to the tragus, to the midline just behind the hairline (Video 3).

**Video 3:** 3D (stereoscopic) stop motion video depicting a pterional approach The skin incision starts at the superior rim of the zygomatic arch and arcs to the midline. The scalp flap, along with the underlying STA, is retracted anteriorly over the orbit until the superior edge of the suprafascial fat pad is observed. Superior to the STL, the frontal pericranium is exposed; inferiorly, the superficial temporal fascia is exposed. Interfascial dissection is performed along the most anterior part of the STL. Subfascial dissection is also performed to dissect both layers of temporal fascia and expose the temporalis muscle. A musculo-fascial cuff is left along the STL. The temporalis muscle is dissected along the STL and reflected anteroinferiorly over the zygomatic arch. The MacCarty keyhole is identified 5-6 mm posterior to the three-junction suture. The second burr hole is drilled above the zygomatic arch in the temporal bone. The frontotemporal craniotomy is carefully performed around the pterion and the bone flap is removed to expose the dura and anterior branch of the middle meningeal artery. A rongeur and drill are used to flatten the lesser wing of sphenoid and expand the subfrontal space. Abbreviations: STA, superficial temporal artery; STL, superior temporal line.

**Figure 3:**
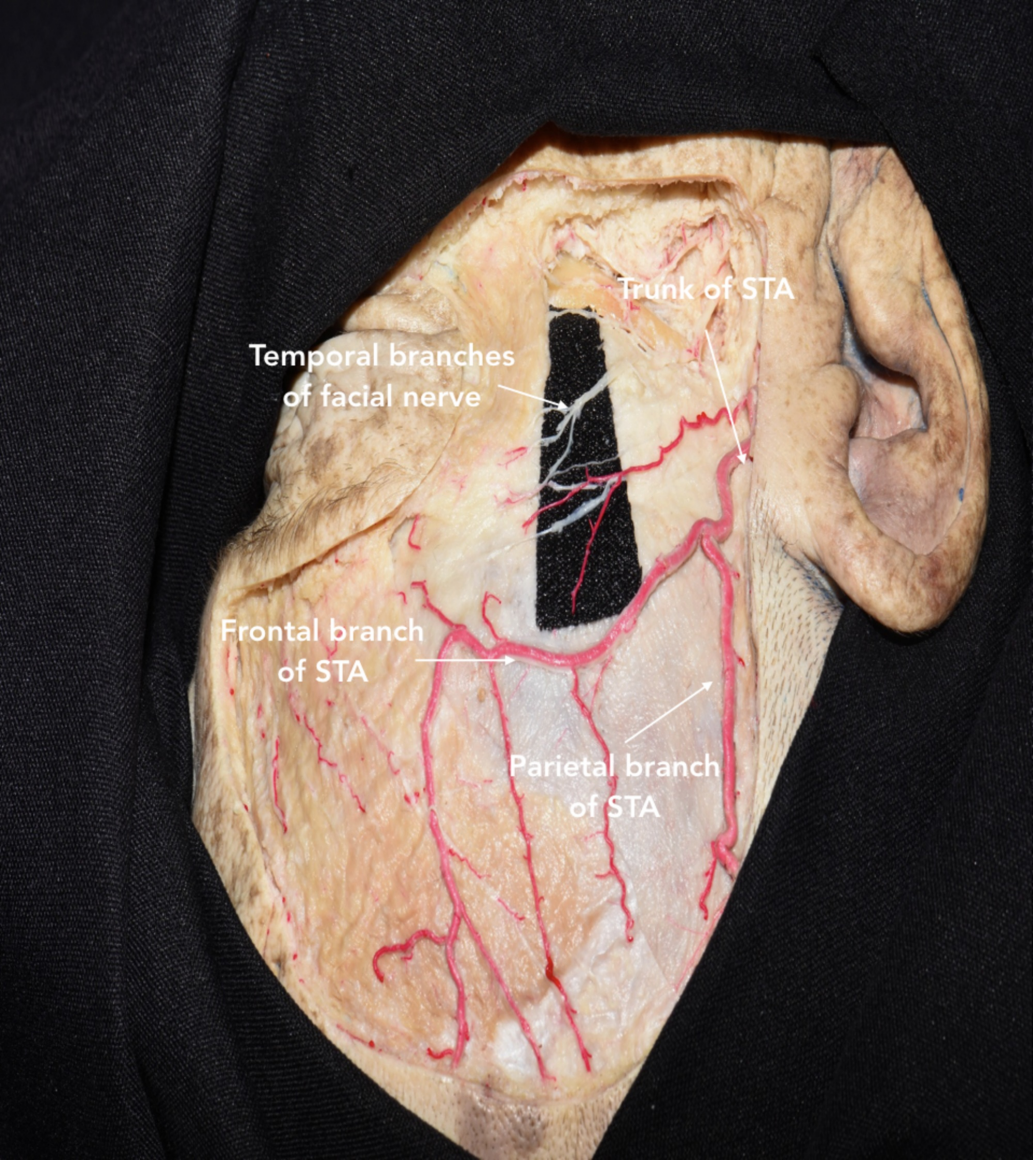
Dissection showing the course of the superficial temporal artery (STA) and temporal branches of the facial nerve (TBFN) The STA should be controlled early in the procedure to reduce bleeding during the skin incision. Moreover, the STA can be used as a landmark for locating the TBFN depending on the bifurcation point of STA relative to the superior orbital rim. In either case, the TBFN has been identified within 1 cm of the frontal branch of STA above the zygomatic arch.

Since the temporal branch of the facial nerve (TBFN) extends above the anterior 2/3 of the zygomatic arch, the incision should not exceed 1 cm anterior to the tragus being that the most posterior branch of the facial nerve would be around 1.5 cm anterior to the tragus (Figure 3) [7]. Care should also be taken to avoid injuring the superficial temporal artery (STA), which can be observed along the incision line.

Dissection of skin flap

The skin and subcutaneous tissue are carefully isolated as a single layer from periosteum, muscle, and fat through a transareolar (subgaleal) dissection from the midline to the inferior portions. The skin flap containing the skin, subcutaneous tissue, galea, and STA is retracted anteriorly toward the orbit until the superior edge of the suprafascial fat pad is observed. Since TBFN are located in this fat pad, loose areolar dissection should not extend inferiorly beyond this point. Specifically, the dissection plane is lost in an area called the inferior temporal septum that extends 3 cm horizontally and 2.3 cm superiorly from the zygomatic arch to the lateral orbital rim (Interactive Model 4) [8].

**Video 4:** Volumetric model of the soft tissue layers relevant to the pterional approach, with labels and annotations Based on the course of TBFN across the STL, the anatomy can be divided into layers superior and inferior to the STL. From superficial to deep, the soft tissue layers inferior to STL are skin, subcutaneous tissue, galea, loose areolar tissue, suprafascial fat pad, superficial temporal fascia, interfascial fat pad, deep temporal fascia, subfascial fat pad, temporalis muscle, and periosteum. The soft tissue layers superior to the STL are distinguished by the absence of temporal fascia and temporalis muscle above the skull. In addition, the periosteum is referred to as the frontal pericranium. TBFN, temporal branches of facial nerve; STL, superior temporal line.

**Figure 4:**
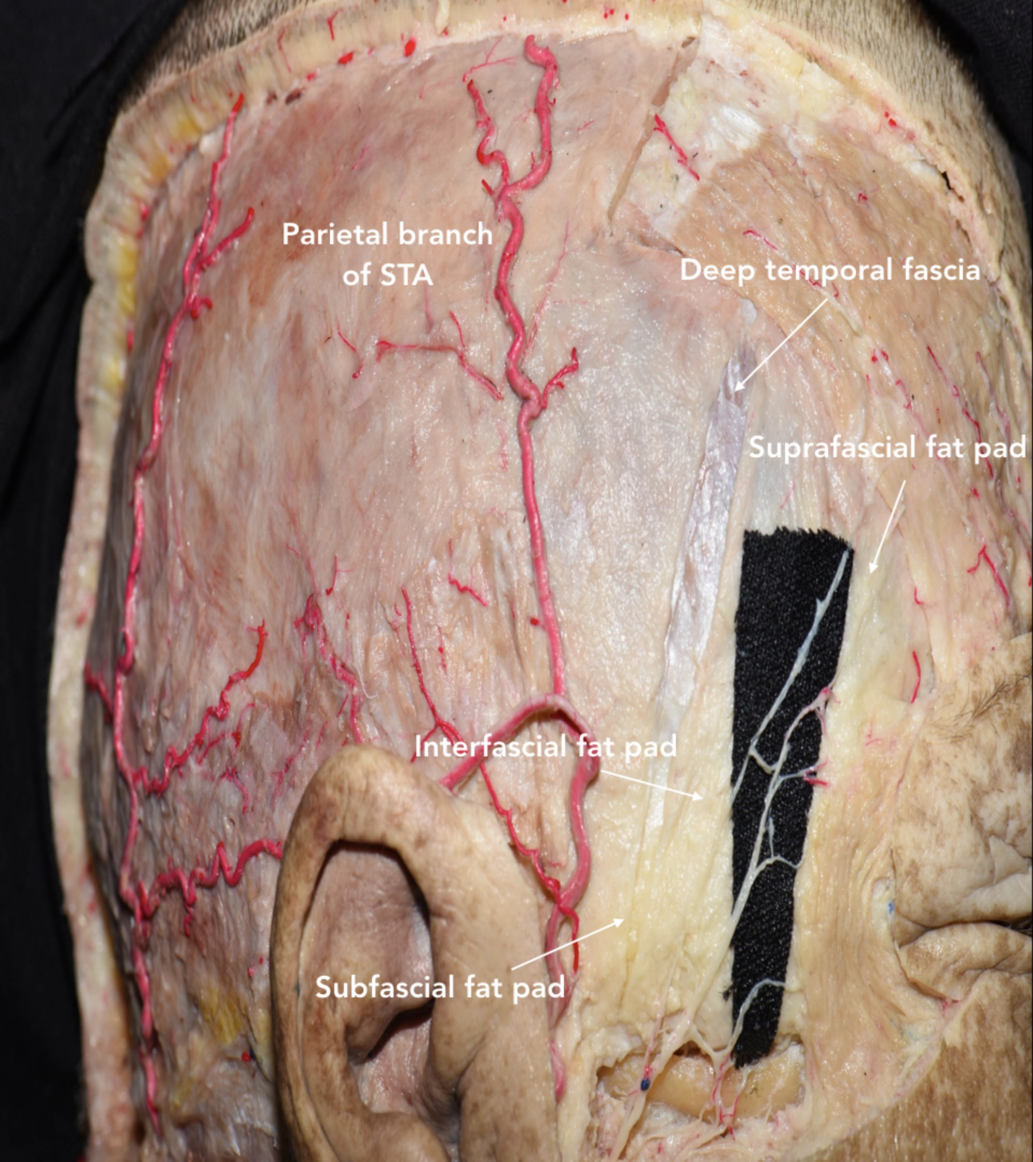
Lateral view of head showing the three fat pads (suprafascial, interfascial, subfascial), galea, deep temporal fascia, and periosteum The TBFN courses through the suprafascial fat pad, and then runs anterosuperiorly toward the forehead. TBFN, temporal branches of facial nerve; STA, superficial temporal artery.

Also, it is important to note that the TBFN pass through different tissue layers within the frontotemporal region. While the TBFN is identified in the suprafascial fat pad at the level of the zygomatic arch, it courses above the galea in the superior aspect of the frontotemporal region (Figure 4) [8].

The mean distance from the TBFN to various landmarks can be referenced to carefully navigate through this region (Table 1).

**Table 1:** Mean distance from various landmarks to the temporal branches of facial nerve (TBFN) Length measurements were adapted from Salas et al. [8].

Landmark	Mean distance from middle branch of TBFN	Range
Superolateral orbital angle	2.6 cm	1.6-3.6 cm
Inferolateral orbital angle	4.32 cm	2.9-5.1 cm
Frontozygomatic suture	3.23 cm	1.8-4.4 cm
Tragus	2.25 cm	1.7-3.2 cm
Lateral canthus	4.0 cm	3.4-4.6 cm
	3.8 cm (anterior branch)	3.4-4.2 cm
	6.0 cm (posterior branch)	5.6-6.4 cm

Dissection of temporal fascia

In the 1980s, Yasargil et al. popularized the interfascial technique for maximally retracting the temporalis muscle without injuring the facial nerves [9]. The superficial temporal fascia is initially incised along the most anterior part of STL, where the superficial temporal fascia and deep temporal fascia are continuous. The interfascial fat pad is observed approximately 4 cm above the lateral orbital rim (Figure 4) [7]. Once the interfascial fat pad and deep temporal fascia are identified, the incision is continued toward the posterior root of the zygomatic arch. The interfascial fat pad is carefully dissected and reflected together with the superficial temporal fascia over the scalp flap. This layer is kept in continuity with the frontal pericranium to preserve TBFN and minimize the risk of post-operative cosmetic defects [2]. The dissection is continued on the deep temporal fascia attached to the outer surface of the temporalis muscle. An additional cut parallel to the surface of the skull is made to release the junction of the superficial temporal fascia and the frontal pericranium from the STL. This interfascial-subpericranial flap can now be reflected anteriorly without injuring the TBFN passing through these layers [7].

As an alternative to the interfascial technique, a subfascial technique has been described where both layers of the temporal fascia are dissected from below the superior temporal line to the posterior root of the zygomatic arch [10]. A musculo-fascial cuff is left along the STL for reattachment of temporalis muscle at the closure. This cuff also improves the cosmetic and functional outcomes of the patient by preventing injury to the TBFN that run through the interfascial fat pad [11]. The dissection plane is maintained between the temporal fascia and muscle fibers of the temporalis muscle to ultimately elevate both layers of the temporal fascia, including the interfascial fat pad, and expose the temporalis muscle. Similar to the interfascial technique, a parallel cut is made to release the junction of the temporal fascia and frontal pericranium from the STL [7]. This subfascial-subpericranial flap can now be reflected anteriorly. Compared to the interfascial technique, the subfascial technique has been noted to be safer and efficient in preserving the TBFN [11,12]. However, care should be taken to separate the deep temporal fascia from the temporalis muscle without damaging the surface of the temporalis muscle.

Furthermore, Coscarella et al. presented the submuscular technique to dissect the muscle and temporal fascia in a single layer off the bone [10]. The dissection is initiated posteriorly to the suprafascial fat pad. This technique avoids the risk of damage to the TBFN, which occasionally crosses the interfascial fat pad before innervating the frontalis muscle. Moreover, this procedure is effective for reaching superficial lesions that do not require significant anterior exposure of the bone. Yet, the single-layer dissection reduces muscle retraction as well as the maneuverability of surgical instruments within the operative corridor. This technique can be combined with a partial subgaleal dissection (behind the suprafascial fat pad) or an osteoplastic exposure.

**Figure 5:**
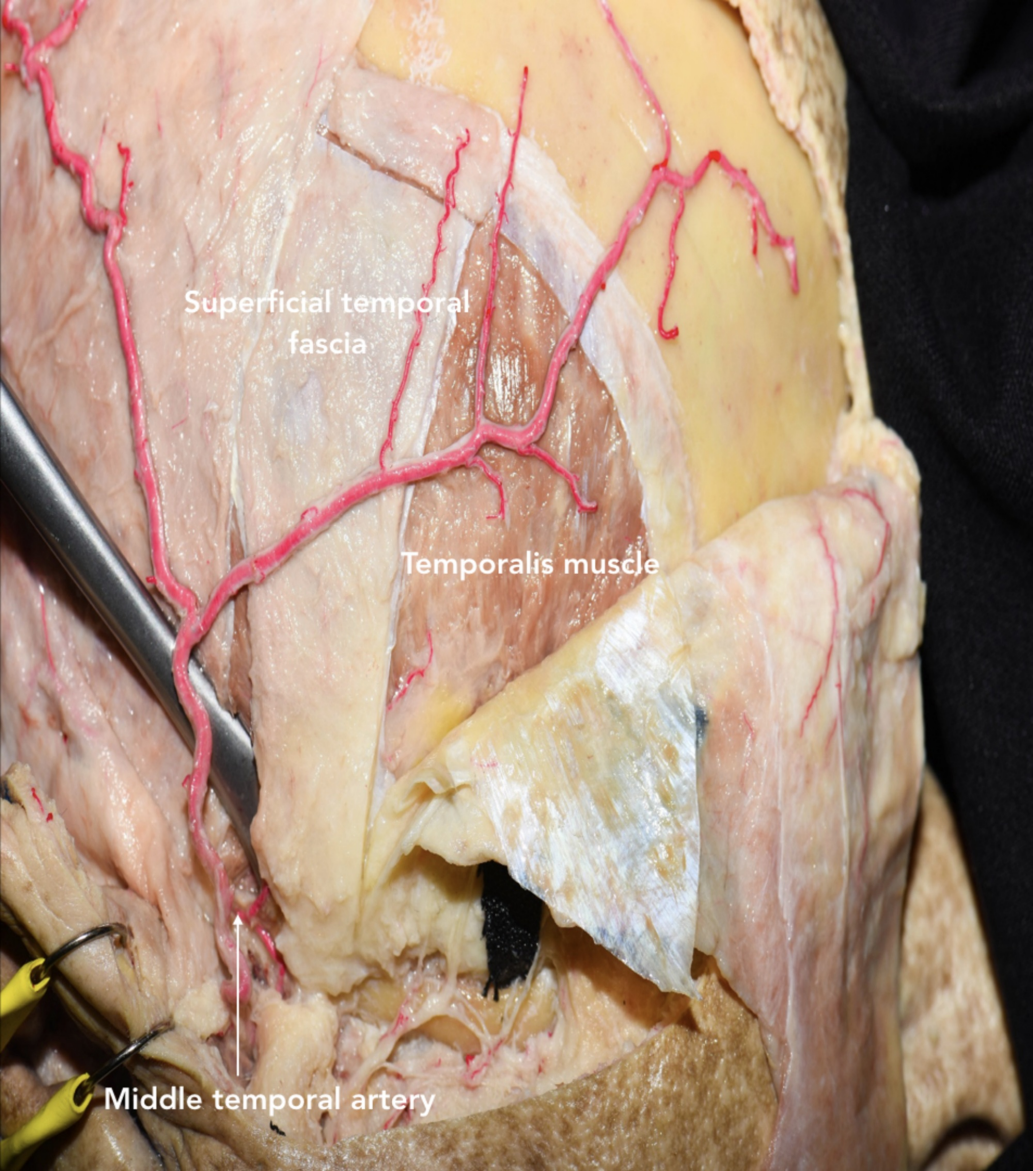
Oikawa technique for dissecting the temporalis muscle inferiorly to superiorly This retrograde dissection runs opposite to the muscle fibers.

After dissecting the temporal fascia, the temporalis muscle is cut and detached from the bone. Two cuts are performed through the Oikawa technique [13]: one is vertical along the STL and skin incision, and the other is transversal 1-2 cm below the STL to generate the musculo-fascial cuff (Figure 5). It is subsequently reflected caudally-to-rostrally over the zygomatic arch to rexztract the temporalis muscle and expose the pericranium maximally. This retrograde dissection allows the muscle to be elevated along its natural plane, minimizes the risk of muscle atrophy, and protects the underlying deep temporal nerves and artery. The muscle is reflected over the zygomatic arch using fish hooks to maximally retract the temporalis muscle and expose the pericranium.

Alternatively, a T-shaped incision could be performed to incise the muscle vertically into two muscle flaps that are reflected anteriorly and posteriorly (Figure 12E). Care should be taken when getting close to the zygomatic root because this is where the middle temporal artery, a proximal branch of STA, is located and could be a source of inadvertent bleeding.

Craniotomy

**Figure 6:**
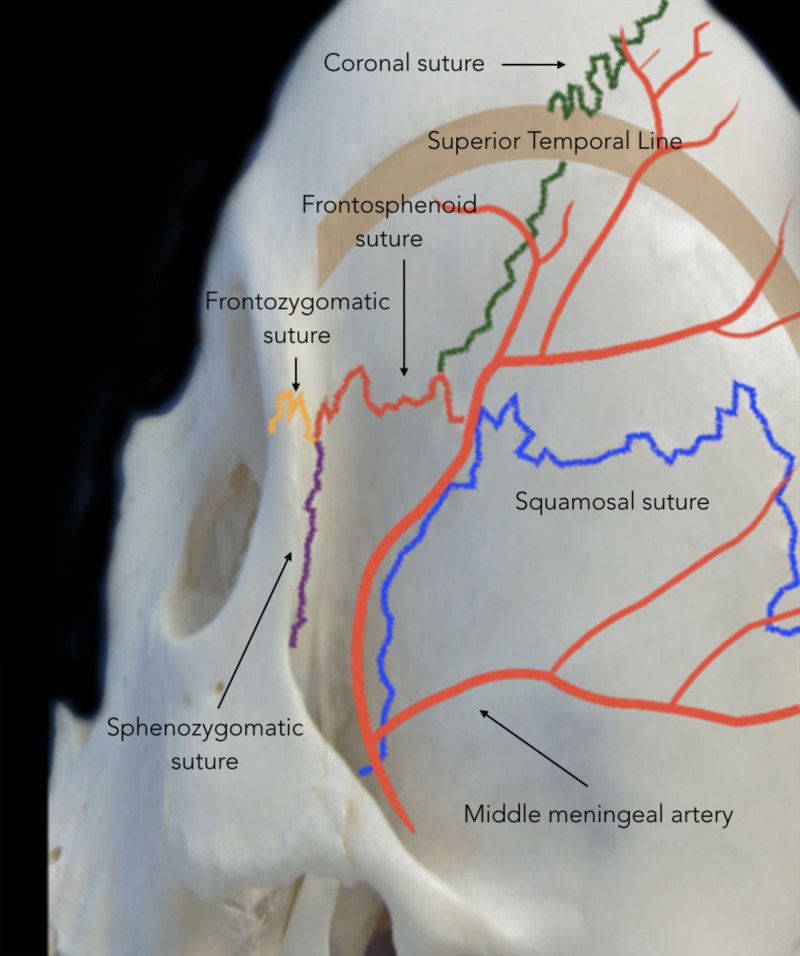
Lateral view of skull showing the course of the middle meningeal artery The anterior branch of the middle meningeal artery runs below the pterion, from the middle cranial fossa and superiorly to the anterior cranial fossa.

**Figure 7:**
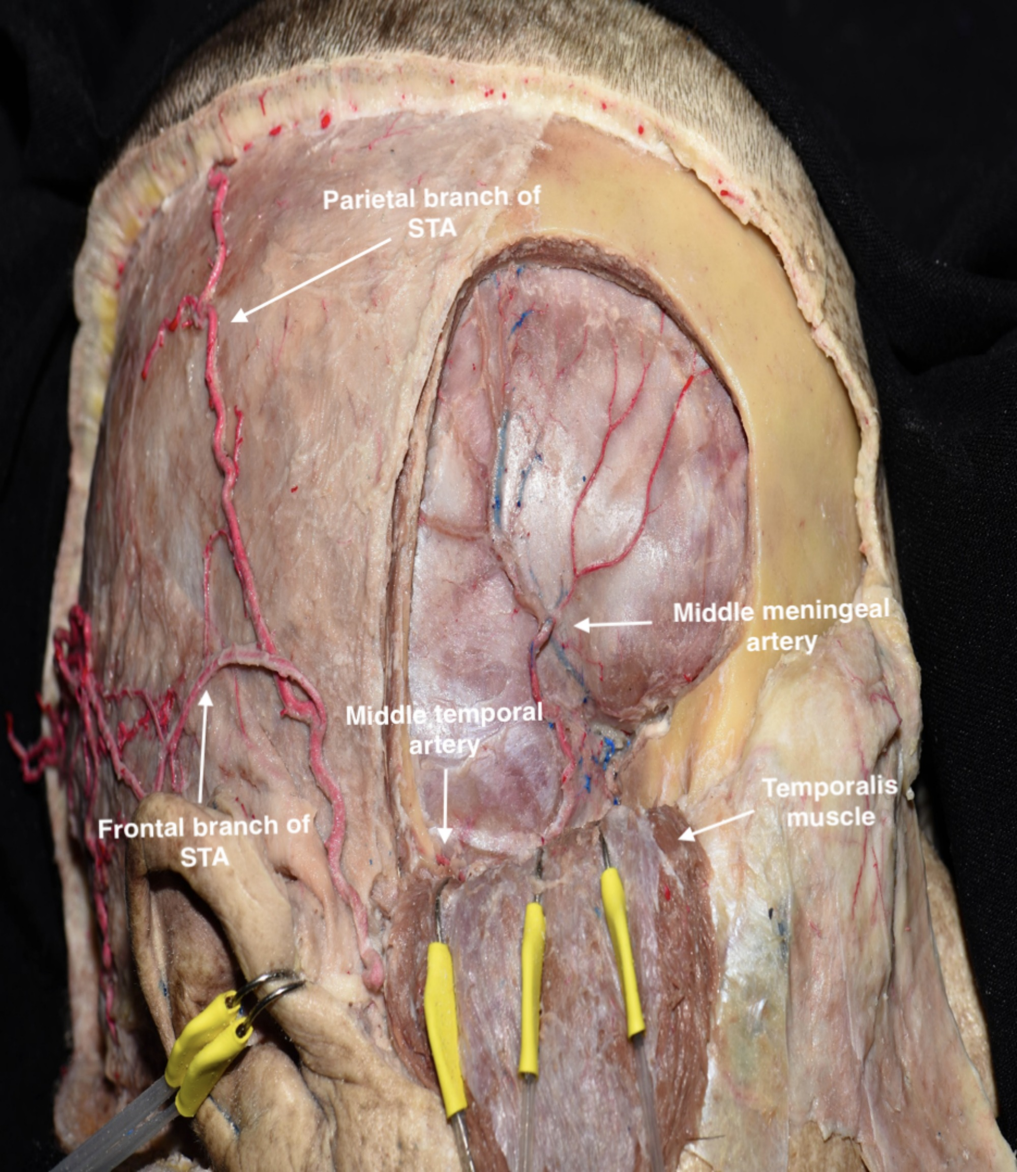
Pterional bone flap was removed to illustrate the course of the anterior branch of the middle meningeal artery below the pterion STA, superficial temporal artery

**Figure 8:**
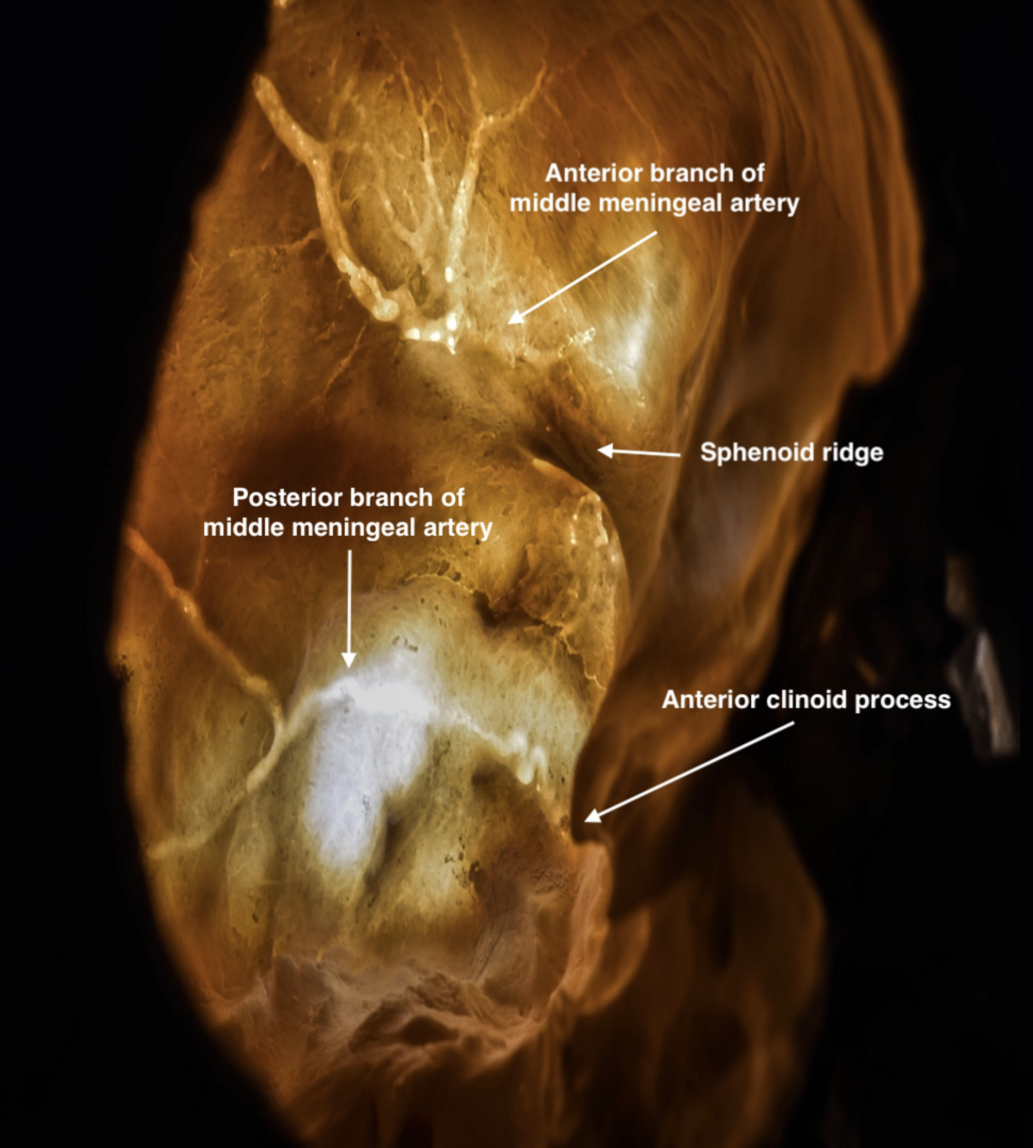
Transillumination of the skull showing the course of the middle meningeal artery The anterior branch is located above the sphenoid ridge; the posterior branch is located below.

**Figure 9:**
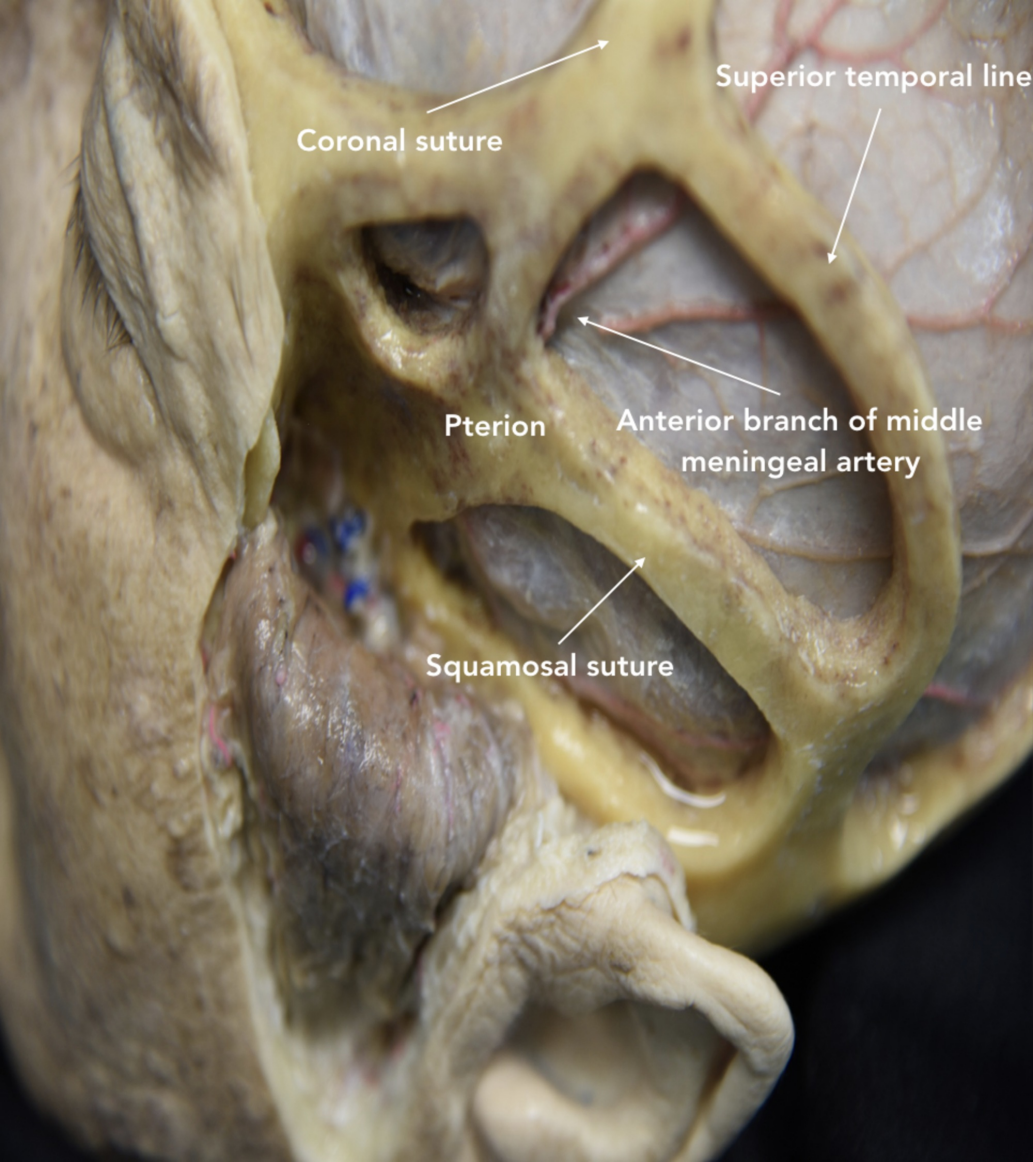
Lateral view showing windows of the pterional approach based on cranial sutures The anterior branch of the middle meningeal artery is observed running superiorly.

Before performing the craniotomy, the surgeon should identify relevant craniometric points that could be used to locate critical structures underneath the bone. The pterion is a major craniometric landmark for the anterior sylvian point and the anterior branch of the middle meningeal artery (Figure 6). It is important to be familiar with the course of the middle meningeal artery to avoid placing a burr hole at the bifurcation point and to know what to coagulate when bleeding occurs (Figure 7-9). Another landmark is the squamous suture, whereby its highest point can be used to locate the inferior rolandic point that lies at the inferior extent of the central sulcus [14].

The first burr hole is drilled along the MacCarty keyhole to expose the floor of the anterior cranial fossa. The keyhole should be positioned along the frontosphenoid suture approximately 5-6 mm posterior to the junction of the frontosphenoid, frontozygomatic, and sphenozygomatic sutures: a landmark also referred to as the three-suture junction (Interactive Model 1) [15]. To avoid orbital opening, the drill should be performed in the posterior angle. The second (temporal) burr hole is drilled above the zygomatic arch in the temporal bone. The third burr hole is drilled along the STL in the most posterior portion of the exposed bone. However, the number of burr holes placed is not fixed and can be tailored according to the underlying pathology and age of the patient. Elderly patients tend to have the dura mater firmly attached to the inner wall of the skull, making it prone to be inadvertently damaged during its exposure.

After detaching the dura from the internal bone, the frontotemporal craniotomy is initiated from the temporal burr hole and directed posteriorly and superiorly. Once the craniotome reaches 1-2 cm above the STL, the craniotomy can be directed anteriorly and then inferiorly to the keyhole. Next, the temporal burr hole is joined with the keyhole until the lesser wing of the sphenoid is reached. According to the surgical target and strategy, the craniotomy can be tailored by increasing or reducing the exposure of the frontal or temporal lobe. To remove the bone flap, the posterior portion of the lesser wing of sphenoid must be drilled. Care should be taken to avoid fracturing the bone flap and tearing the dura, particularly along the inferior area. Finally, the bone flap is removed to expose the dura and SF (Interactive Model 5).

**Video 5:** Volumetric model of dural exposure following a pterional craniotomy, with labels and annotations Emphasis is placed on the anterior branch of the middle meningeal artery, which runs beneath the pterion and must be carefully preserved. The Sylvian fissure is exposed along with the following cortical structures: inferior frontal gyrus, superior temporal gyrus, and parts of the middle frontal gyrus and middle temporal gyrus.

Drilling of basal structures

**Figure 10:**
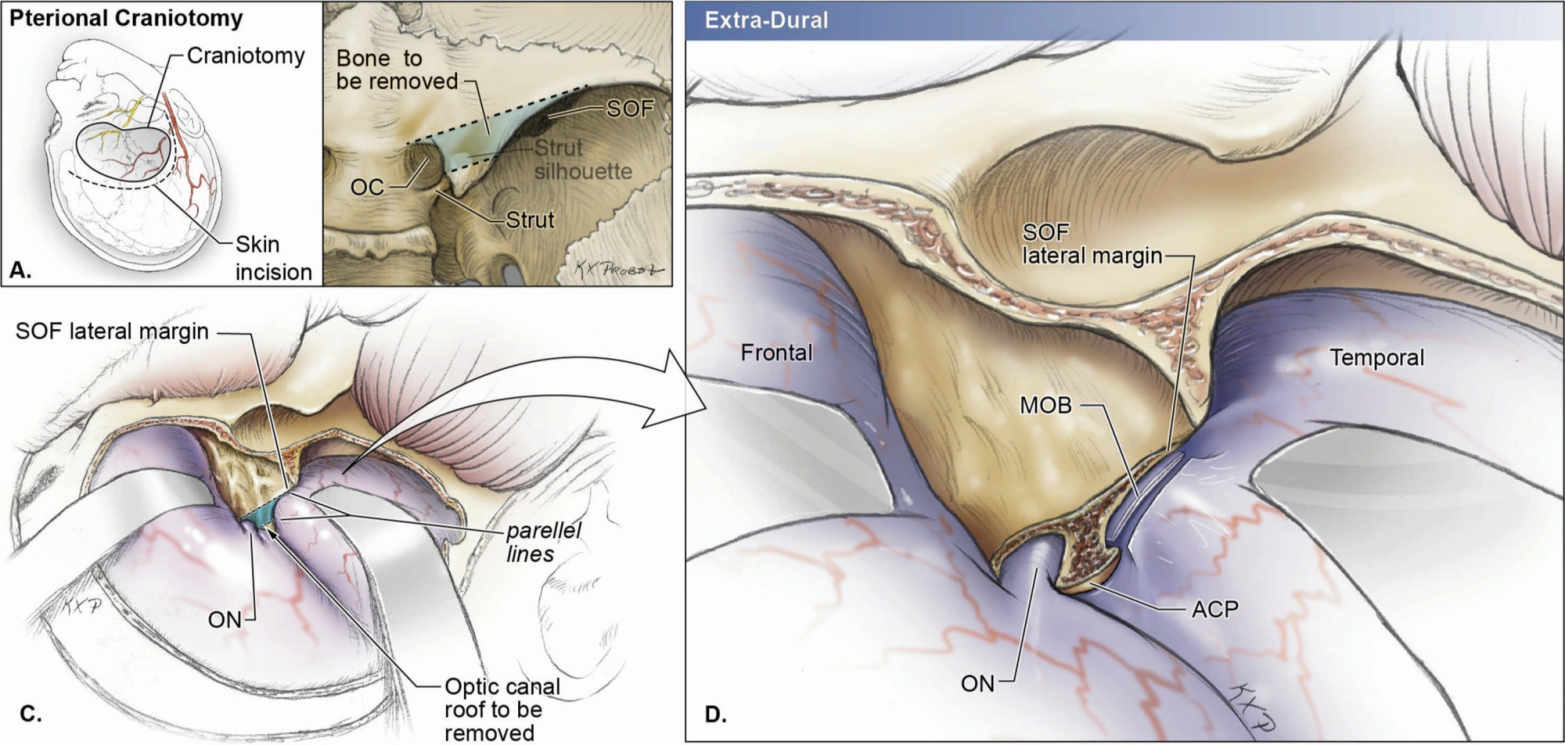
Schematic drawings of a right pterional approach with extra-dural removal of the anterior clinoid process A) Schematic drawing of the skin incision and craniotomy of the pterional approach. Care should be taken to avoid injuring the anterior branch of the middle meningeal artery and temporal branches of the facial nerve. B) The blue shaded area shows the part of the optic canal roof that must be removed if an extension of drilling the lesser wing of sphenoid or extra-dural anterior clinoidectomy is necessary. OC, optic canal; SOF, superior orbital fissure. C) Intraoperative view after the pterional craniotomy. The blue shaded area (part of the optic canal roof) must be removed for further medial exposure. ON, optic nerve; SOF, superior orbital fissure. D) Intraoperative view after part of the optic canal roof is removed. The meningo-orbital band (MOB) connects the periosteal layer of the frontotemporal basal dura to the periosteal layer of the periorbita and demarks the transition between the medial and lateral portions of the middle fossa. By partially removing part of the optic canal roof and separating the dura propria of temporal lobe from the inner cavernous membrane, the MOB can be safely detached from the periorbita to expose the middle cranial fossa, including the anterior clinoid process. ACP, anterior clinoid process; MOB, meningo-orbital band; ON, optic nerve; SOF, superior orbital fissure.

The dura should be detached from the orbital roof, the lesser wing of the sphenoid, and remaining temporal squama. These structures can then be drilled to flatten the bone and expand the operative corridor connecting both anterior and middle cranial fossae that results in a decrease on retraction of intradural structures and an increase in the surgical angles. However, there are two main caveats to drilling these areas. First, the orbital roof is a relatively thin layer of bone that can be easily fractured with the drill or during dissections. Disruption into the orbit may lead to prolapse of the orbital fat, which will require reconstruction of the periorbita. Second, the medial limit of drilling the lesser wing of the sphenoid is the meningo-orbital band (MOB) that lies superolateral to the superior orbital fissure and demarks the transition between the medial and lateral portions of the middle fossa. The MOB connects the periosteal layer of the frontotemporal basal dura to the periosteal layer of the periorbita [16]. If an extension of drilling the lesser wing of sphenoid or epidural anterior clinoidectomy is necessary, then the surgeon should first locate and coagulate the meningo-orbital artery that lies within the MOB [17]. By partially removing the lateral wall of the superior orbital fissure and separating the dura propria of temporal lobe from the inner cavernous membrane, the MOB can be safely detached from the periorbita to expose the middle cranial fossa, including the anterior clinoid process (Figure 10A-D). These steps will reduce the risk of injury to surrounding cranial nerves and vascular structures, including the superolateral portion of the cavernous sinus, the optic nerve, and internal carotid artery.

Dural opening

**Figure 11:**
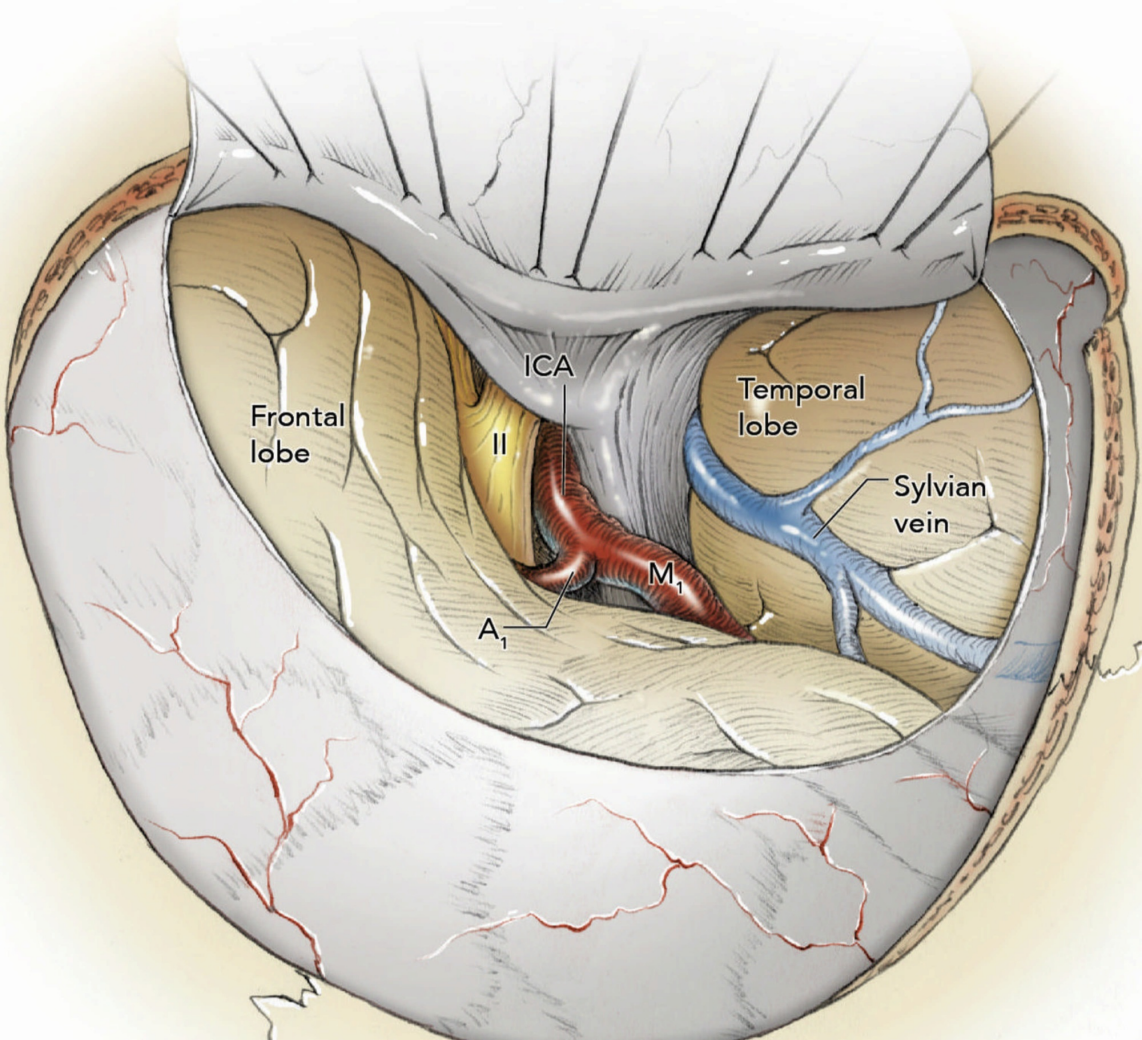
Intraoperative view after the dural opening The Sylvian fissure is exposed along with the optic nerve, Sylvian vein, internal carotid artery, and the first segments of the anterior cerebral artery and middle cerebral artery. A1, horizontal segment of anterior cerebellar artery; ICA, internal carotid artery; II, optic nerve; M1, horizontal segment of middle cerebral artery.

Dural suspension around the craniotomy should be performed to avoid blood draining into the surgical field and postoperative epidural hematomas. Usually, a C-shaped incision is performed to open the dura with the convexity facing the posterior portion of the craniotomy. An additional cut in the middle of the C and parallel to SF can be made to avoid roughness or superimposition. After the dural opening, SF is exposed along with the optic nerve, Sylvian vein, internal carotid artery, and the horizontal segments of the anterior cerebral artery and middle cerebral artery (Figure 11). The following cortical structures are also exposed from superior to inferior: the inferior portion of the middle frontal gyrus, inferior frontal gyrus, superior temporal gyrus, and middle temporal gyrus.

## Discussion

Modifications of the Standard Pterional Approach

Mini-pterional approach

The pterional craniotomy has undergone several modifications over time to improve the safety and efficiency of reaching lesions in the supratentorial region [1]. One of the latest modifications includes the mini-pterional (MPt) craniotomy, where a curvilinear incision is made from 1.0 cm above the zygomatic arch to the ipsilateral midpupillary line (Video 4) [18].

**Video 4:** 3D (stereoscopic) stop motion video depicting a mini-pterional approach A curvilinear incision is made from the zygomatic arch to the ipsilateral midpupillary line. The superficial temporal fascia is dissected and elevated to expose the temporalis muscle. The temporalis muscle is dissected and reflected anteroinferiorly over the zygomatic arch. A single burr hole is drilled above the zygomatic arch, and a craniotomy is performed to expose the dura. The lesser wing of sphenoid is drilled to expand the subfrontal space. An extra-dural anterior clinoidectomy is also performed to increase the surgical space medially. A C-shaped incision is performed to open the dura and expose the Sylvian fissure.

This approach aims to reduce the extent of craniotomy without compromising surgical exposure and working angles (Interactive Model 6). By tightening the surgical corridor on SF, the MPt approach is optimal for unruptured middle cerebral artery, internal carotid artery, and ophthalmic artery aneurysms [19]. In addition, the MPt approach significantly reduces the extent of temporalis muscle dissection. This change decreases surgical time and improves cosmetic outcomes, particularly for older patients [18]. Nevertheless, limitations of the MPt approach include reduced maneuverability of surgical instruments and diminished exposure of distal middle cerebral artery and anterior cerebral artery aneurysms.

**Video 6:** Volumetric model of the mini-pterional (MPt) approach, with labels and annotations The medial limit of the skin incision is the ipsilateral midpupillary line.

Two-part pterional craniotomy

**Figure 12:**
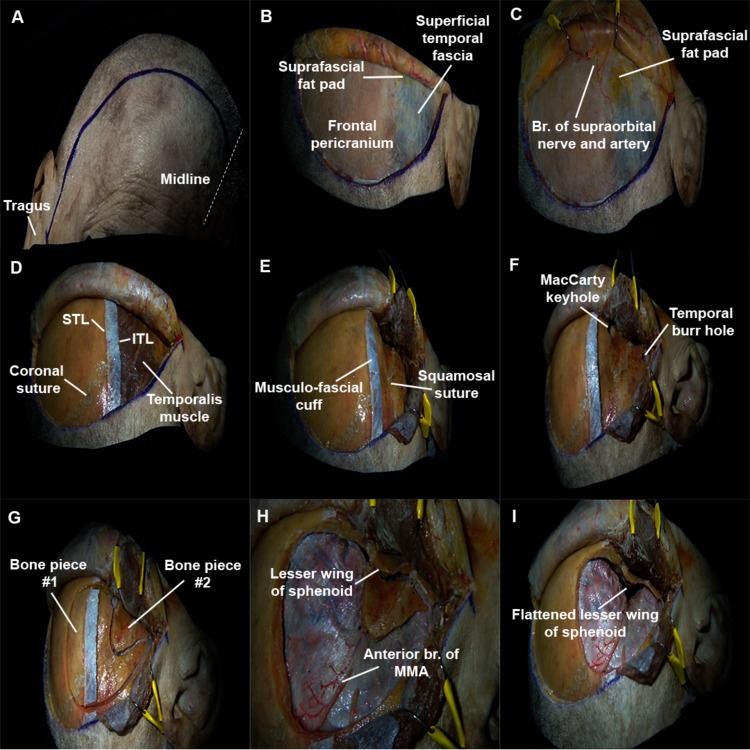
Two-part pterional approach A) Marking starts at the superior rim of the zygomatic arch, 1 cm anterior to the tragus, and extends to the midline. B) The scalp flap is reflected over the orbit to expose the superficial temporal fascia (inferior to STL) and frontal pericranium (superior to STL). STL, superior temporal line. C) The scalp flap is further retracted to show the suprafascial fat pad. Incisions should not extend beyond the dotted line to avoid injuring the temporal branches of facial nerve. Br, branches. D) The frontal pericranium and both layers of the temporal fascia are elevated together to maintain their continuity over the STL. A musculo-fascial cuff is left along the STL. ITL, inferior temporal line; STL, superior temporal line. E) The temporalis muscle is dissected via a T-shaped incision to produce two muscle flaps. F) The first burr hole is drilled behind the MacCarty keyhole towards the anterior cranial fossa. The second burr hole is placed in squamous portion of the temporal bone. G) V-shaped craniotomy is performed around the lateral sphenoid wing to create two bone pieces. H) The second bone cut is made with a footplate attachment passing under the lesser wing of sphenoid. Br, branch; MMA, middle meningeal artery. I) Result of the two-part pterional craniotomy that shows the expanded subfrontal space.

A second modification includes a two-part pterional craniotomy, which facilitates the reconstruction of the lesser wing of sphenoid (Figure 12A-I; Interactive Model 7) [20]. Initially, two burr holes are created: the frontal burr hole is created posteroinferiorly to the MacCarty keyhole and the second burr hole is created posteriorly in the squamous portion of the temporal bone. Starting at the temporal burr hole, a curvilinear craniotomy is performed toward the supra-orbital margin. From the frontal burr hole, the cut is made superiorly toward the supra-orbital margin, as well as inferiorly around the lesser wing of sphenoid toward the temporal burr hole. Once the first part of the bone piece is removed, extradural dissection is performed to expose the lesser wing of sphenoid deep to the superior orbital fissure and separate the dura from the floors of the anterior and middle cranial fossa. The second bone piece is removed with a foot plate attachment to preserve the lateral sphenoid wing. During the closure, the two bone parts are connected with titanium plates. These steps eliminate the need to repair damages to the sphenoid wing, which contributes to improved cosmetic results for the patient. Although several other modifications to the standard PA are presented in the literature, a craniotomy should be carefully selected and tailored according to the patient’s pathology and unique anatomy.

**Video 7:** Volumetric model of the two-part pterional approach, with labels and annotations This modified approach avoids drilling the lateral sphenoid wing by performing the craniotomy in two pieces.

## Conclusions

Understanding the 3D relationship of the anatomical structures found during a fronto-temporo-sphenoidal exposure is paramount to creating safe surgical corridors while using PA or any of its variations. The PA is one of the most common and flexible approaches in neurosurgery, and it has quickly evolved with the advent of new neurosurgical technologies and the experience that surgeons have acquired throughout dealing with an immense variety of pathologies. The present study illustrates the major steps of the PA through the use of 3D technologies to facilitate a precise understanding of the layer-by-layer surgical anatomy of PA. In particular, the course of TBFN must be carefully examined to avoid injuring the nerve during interfascial, subfascial, or submuscular dissections. A clear topographical comprehension of the pterion, the lesser sphenoid wing, and the superior orbital fissure is essential in order to significantly expand the intracranial corridor with minimal brain retraction during later stages of PA. Medical applications of computer graphics are exponentially advancing and becoming a critical part of our decision-making workflow, surgical planning, and anatomical education curriculum. The interactive models presented in this article allow for clear visualization of the surgical anatomy and windows in 360-degrees and VR. This new modality of recording neuroanatomical dissections renders a closer look at every nuance of the topography experienced by our team in the laboratory. By accurately depicting essential landmarks, stereoscopy and VM can be valuable resources for anatomical education and surgical planning.

## References

[1] Altay T, Couldwell WT (2012). The frontotemporal (Pterional) approach: an historical perspective. Neurosurgery.

[2] Yaşargil MG (1984). Microneurosurgery.

[3] Lawton MT (2011). Seven aneurysms: tenets and techniques for clipping.

[4] Samson DS, Hodosh RM, Clark WK (1978). Microsurgical evaluation of the pterional approach to aneurysms of the distal basilar circulation. Neurosurgery.

[5] Meybodi AT, Lawton MT, Rubio RR, Yousef S, Benet A (2018). Contralateral approach to middle cerebral artery aneurysms: an anatomical-clinical analysis to improve patient selection. World Neurosurg.

[6] Wen HT, Rhoton AL, De Oliveira E, Castro LHM, Figueiredo EG, Teixeira MJ (2009). Microsurgical anatomy of the temporal lobe: Part 2-sylvian fissure region and its clinical application. Neurosurgery.

[7] Poblete T, Jiang X, Komune N, Matsushima K, Rhoton AL (2015). Preservation of the nerves to the frontalis muscle during pterional craniotomy. J Neurosurg.

[8] Salas E, Ziyal IM, Bejjani GK, Sekhar LN (1998). Anatomy of the frontotemporal branch of the facial nerve and indications for interfascial dissection. Neurosurgery.

[9] Yaşargil MG, Reichman MV, Kubik S (1987). Preservation of the frontotemporal branch of the facial nerve using the interfascial temporalis flap for pterional craniotomy. J Neurosurg.

[10] Coscarella E, Vishteh AG, Spetzler RF, Seoane E, Zabramski JM (2000). Subfascial and submuscular methods of temporal muscle dissection and their relationship to the frontalis branch of the facial nerve. J Neurosurg.

[11] Spiriev T, Poulsgaard L, Fugleholm K (2015). Techniques for preservation of the frontotemporal branch of facial nerve during orbitozygomatic approaches. J Neurol Surg B.

[12] Tayebi Meybodi A, Lawton MT, Yousef S, Sánchez JJG, Benet A (2017). Preserving the facial nerve during orbitozygomatic craniotomy: surgical anatomy assessment and stepwise illustration. World Neuros.

[13] Oikawa S, Mizuno M, Muraoka S, Kobayashi S (1996). Retrograde dissection of the temporalis muscle preventing muscle atrophy for pterional craniotomy. J Neurosurg.

[14] Ribas GC, Yasuda A, Ribas EC, Nishikuni K, Rodrigues AJ (2006). Surgical anatomy of microneurosurgical sulcal key points. Neurosurgery.

[15] Shimizu S, Tanriover N, Rhoton AL, Yoshioka N, Fujii K (2005). MacCarty keyhole and inferior orbital fissure in orbitozygomatic craniotomy. Neurosurgery.

[16] Froelich SC, Aziz KMA, Levine NB, Theodosopoulos PV, Van Loveren HR, Keller JT (2007). Refinement of the extradural anterior clinoidectomy: surgical anatomy of the orbitotemporal periosteal fold. Neurosurgery.

[17] Chaddad-Neto F, Campos-Filho JM, Dória-Netto HL, Faria MH, Ribas GC, Oliveira E (2012). The pterional craniotomy: tips and tricks. Arq Neuro-Psiquiatr.

[18] Figueiredo EG, Deshmukh P, Nakaji P, Crusius MU, Crawford N, Spetzler RF, Preul MC (2007). The minipterional craniotomy: technical description and anatomic assessment. Neurosurgery.

[19] Caplan JM, Papadimitriou K, Yang W (2014). The minipterional craniotomy for anterior circulation aneurysms: Initial experience with 72 patients. Neurosurgery.

[20] Devasagayam S, Benet A, McDermott MW (2012). Two part pterional craniotomy: technical note. Cureus.

